# Nutritional status and associated factors among adolescents in Senegal: a nationwide cross-sectional study

**DOI:** 10.1136/bmjopen-2024-095574

**Published:** 2025-10-28

**Authors:** Mariame Sy, Adama Diouf, Abdou Badiane, Maty Diagne Camara, Abdoulaye Diagne, Nicole Idohou-Dossou

**Affiliations:** 1 Laboratoire de Recherche en Nutrition et Alimentation Humaine, Faculté des Sciences et Techniques, Université Cheikh Anta Diop, Dakar, Senegal; 2 Division de l’Alimentation et de la Nutrition, Direction de la Santé de la mère et de l’Enfant, Government of Senegal Ministry of Health and Social Action, Dakar, Senegal; 3 Consortium pour la Recherche Economique et Sociale, Dakar, Senegal

**Keywords:** Adolescent, NUTRITION & DIETETICS, PUBLIC HEALTH

## Abstract

**Abstract:**

**Objective:**

Adolescents are a vulnerable group as they undergo rapid physical changes that can impact their nutritional status. Despite the implementation of some interventions addressing adolescent nutrition in Senegal, there remains a lack of comprehensive data on their nutritional status. This study aimed to assess nutritional status and associated factors among Senegalese adolescents aged 10–19 years.

**Design:**

This cross-sectional study was part of the national food consumption survey, which was conducted among children aged 24–59 months, adolescents aged 10–19 years and adults aged 20 years and older.

**Setting:**

The study was conducted in Senegal at the national level, in rural and urban areas, from July to November 2021.

**Participants:**

1433 adolescents, boys and girls aged 10–19 years were randomly selected from 1800 households in 150 census districts.

**Outcome measures:**

Nutritional status was assessed using body mass index for age z-score and waist-to-height ratio through anthropometric measurements. Dietary practices, health and socioeconomic and sociodemographic data were collected using questionnaires. Data were weighted for representativeness and logistic regression models were used to identify factors associated with underweight, overweight and obesity.

**Results:**

Among the 1433 adolescents, one in five (21.7%) were underweight, 5.9% were overweight, 1.8% had obesity and 5% had abdominal obesity. Male gender (OR=1.74; p<0.001), being in the 10–14 years age group (OR=1.44; p<0.05), having diarrhoea (OR=4.86; p<0.05), skipping lunch daily (OR=2.28; p<0.01) and snacking (OR=1.51; p<0.05) were associated with increased risk of being underweight. Female gender was a predictor of being overweight (OR=4.68; p<0.001) and having abdominal obesity (OR=5.28; p<0.001). Urban adolescents were 59% more likely to be obese than rural adolescents (p<0.05). Adolescents who consumed dietary supplements had greater odds of obesity (OR=3.89; p<0.05) and those who skipped breakfast daily were 4.5 times more at risk of having abdominal obesity. More than 90% of adolescents had three main meals per day. Over 60.3% of adolescents reported snacking and 55.4% of them did so at least once per day. 72% of Senegalese adolescents met the minimum dietary diversity and the mean Dietary Diversity Score was 5.23±1.28.

**Conclusion:**

Underweight is high in Senegalese adolescents, particularly in rural areas and among boys and adolescents aged 10–14 years. Overweight and abdominal obesity among girls and urban adolescents require particular attention. Tackling the double burden of malnutrition in Senegalese adolescents requires a greater consideration of adolescents in policies and strategies, including the implementation of a malnutrition management programme as well as the promotion of healthy diets.

**STRENGTHS AND LIMITATIONS OF THIS STUDY**

- This national study investigated the prevalence and associated factors of the double burden of malnutrition in Senegalese adolescents considering age group and gender.
- The large sample size and representativeness for Senegalese adolescents at the national level, and consideration of the areas of residence, are the main strengths of the study.
- The cross-sectional design limits the ability to investigate the causes behind the identified associations.

## Introduction

### Background

 The WHO defines adolescence as the period between 10 years and 19 years characterised by a high rate of growth and significant psychological changes.^1^ This transitional period between childhood and adulthood is often called an ‘added window of opportunity’ for life cycle growth and development.^2 3^ At this stage of life, adolescent nutritional requirements are significantly increased and changes in body compositions are notable, making adolescents particularly vulnerable to malnutrition.^4^ Many countries are facing the double burden of malnutrition among adolescents, particularly in low and middle-income regions.

Globally, in 2019, underweight affected 10.3% of adolescents^5^ and has slightly decreased since 2010 in both boys and girls,^6^ while overweight and obesity have increased worldwide from 14.4% to 20.2% in boys and from 13.8% to 18.4% in girls between 2010 and 2019.^6^ In Africa, 10.3% of adolescents are underweight.^5^ Moreover, recent trends highlight increasing prevalence of overweight among boys (6.5%) and girls (4.7%), as well as obesity in boys (2.5%) and girls (3.9%).^7^

Malnutrition during adolescence can result in decreased mental capacities and school attendance.^8^ Poor nutritional status, particularly among adolescent girls, can negatively affect reproductive health outcomes and sustain the intergenerational cycle of malnutrition, leading to consequences such as low birth weight. Undernutrition during adolescence not only impairs an individual’s working capacity in adulthood but also undermines the future socioeconomic development of a nation. Both undernutrition and overnutrition during adolescence can severely compromise quality of life and survival, with notable economic repercussions and increased mortality.^9 10^ The global rise in adolescent obesity could be attributed to unhealthy dietary practices.^11 12^ Indeed, urbanisation characterised by a nutritional transition has the potential to expose adolescents to unhealthy dietary patterns, such as high consumption of saturated fats, sugars, salt and ultraprocessed foods.^13 14^

In Senegal, adolescent nutrition has been relatively neglected. Most nutrition programmes or interventions have prioritised children under 5 years of age and women of reproductive age, with little focus on adolescents. Despite the inclusion of girls aged 15–19 years within the women of reproductive age, little attention has been given to the nutritional knowledge and status of adolescents. Interventions targeting adolescents are more focused on reproductive health. Indeed, the country has aligned its priorities and interventions with global challenges, primarily targeting women and children for several decades, while adolescent nutrition has only recently gained attention but remains insufficiently addressed. Therefore, there is no national programme for managing adolescent malnutrition.^15 16^ Despite the implementation of some nutritional interventions such as iron and folic acid supplementation and deworming at school, and while a few small studies have been conducted, no study has been carried out to assess the prevalence and determinants of malnutrition among adolescents at the national level. Those studies have mostly been conducted in Dakar^17 18^ and revealed 0.7% obesity among schoolchildren aged 5–17 years and 29.6% underweight.^19^ Most of the studies include only the 15–19 years age group, as they are usually conducted among women of reproductive age. Moreover, most of the studies did not address dietary patterns and health status among adolescents, making it difficult to explore the whole adolescence target group findings.^15 20^

To effectively address adolescent malnutrition, there is an urgent need to provide evidence-based data to guide decision-making and to accelerate progress towards global goals.

### Objectives

This study aimed to assess the nutritional status and its underlying factors among adolescents in Senegal.

## Methods

### Study design and setting

This nationwide cross-sectional study was part of the food consumption survey conducted among adolescents 10–19 years, children 24–59 months, and adults 20 years and over from July to November 2021. A probabilistic sampling method was employed using a three-stage stratified design. Census districts (CDs) were selected as the primary sampling units. In the second stage, 12 households with at least one adolescent were randomly selected within each CD using a sampling interval of 10. In the third stage, one adolescent was randomly selected from the eligible adolescents living in selected households. This multistage sampling approach allowed the representation of adolescents across diverse geographical, cultural and socioeconomic contexts in Senegal. A total of 150 CDs were randomly selected from urban (91 CDs) and rural (59 CDs) areas of residence. The number of households to be enrolled was determined considering the proportion of children as the rarest target group. A total of 1800 households, including 1091 in urban areas and 709 in rural areas, were therefore targeted.

Before conducting the survey, all the local authorities were informed and an awareness campaign was carried out in the targeted areas. The potentially eligible households were contacted through the community health workers with the support of local and health authorities. In cases where selected households declined participation, they were systematically replaced, with the replacement units drawn strictly from within the same CD. To ensure full consistency with the original design, the identical sampling interval used in the primary selection process was applied during substitution.

### Eligibility criteria

Adolescents, boys and girls, in and out of school and who were apparently healthy were enrolled, except those with mental and physical disabilities for anthropometric measures.

### Data sources/measurement

Two structured questionnaires were administered to the household’s head and adolescents or their tutors. The questionnaires were implemented on a Computer Assisted Personal Interviewing (CAPI) application developed under CSPro Software V.7.5 (United States Census Bureau, USA). At household level, socioeconomic data such as household expenditures were collected to define the household’s economic status (wealth quintile) based on five categories (lowest, second, intermediate, fourth and highest). Among the adolescents, the questionnaire focused on health data particularly on the adolescent’s sociodemographic characteristics, illnesses during the last month before the survey, dietary diversity and habits.

### Dietary diversity and habits

Data on dietary habits were collected to identify eating behaviours such as mealtimes and frequency of consumption, snacking and dietary supplement intake. Additionally, foods consumed over the 24-hour recall were used to define the Dietary Diversity Score (DDS). According to the Food and Agriculture Organization recommendations, since adolescent girls aged 15–19 years are considered in the 15–49 years age group of women of reproductive age, the 10 food groups were used to calculate their DDS. For the other adolescents consisting of boys aged 10–19 years and girls aged 10–14 years, the 9 food groups were used to assess the DDS for individuals.^21 22^

The DDS for all adolescents was classified as adequate (minimum DDS) and inadequate based on a cut-off of five food groups.

### Anthropometry

Weight, height and waist circumference (WC) were measured among adolescents using standard procedures.^23^ Body weight was measured with a very light outfit using an electronic scale with a maximum range of 150 kg (Seca 874, Gmbh & Co, Hamburg, Germany). Height was measured standing on a portable stadiometer (Seca 216, Gmbh & Co, Hamburg Germany). A long inextensible tape was used to measure WC. All measurements were performed in duplicate. Adolescents’ nutritional status was defined based on body mass index for age (BMI-for-age) z-score and waist-to-height ratio (WHtR). Prevalence of underweight, overweight and obesity was determined using the WHO growth reference.^24^ WHtR greater than or equal to 0.5 was used to define abdominal obesity.^25 26^

### Bias

Measurement errors were first minimised during the preparatory phase through comprehensive enumerator training, which included a standardisation session to ensure the accuracy of anthropometric measurements. In addition, a pilot study was conducted to test and refine data collection tools and procedures. During data collection, errors were further minimised through close supervision. Data collected by well-trained enumerators (lead and assistant measurers) were checked in the field by team supervisors to ensure compliance with standard procedures. Moreover, daily feedback was provided, allowing for the correction of identified errors before leaving the CDs. Adolescents who had missing data were also excluded from the statistical analysis.

### Study size

The sample size was calculated based on the Deitchler *et al* methodology using a simulation of nutrient intakes in middle-income countries.^27^ The sampling approach was based on the target group rather than the household, since the food consumption survey focused on three target groups including adolescents’ group. A minimum random sample of 200 respondents, adjusted for design effect and non-response, was considered sufficient to estimate most nutrients with acceptable precision. Using a design effect of 2 and a 5% non-response rate, a minimum of 420 adolescents per area of residence was required.

A non-response rate of 5% was adopted, as recommended by the National Agency for Statistics and Demography, which reported that non-biological studies conducted in Senegal typically achieve response rates of approximately 95%–98%. Considering the estimated proportion of Senegalese adolescents (0.8), with respective proportions of 0.6 and 0.75 for urban and rural areas, a final sample size of 1199 adolescents was determined. In cases of absence or refusal, adolescents were systematically replaced by others randomly selected from the list of eligible adolescents within the same households.

### Quantitative variables

Age was categorised to create new categorical variables: age group and stage of adolescence. Weight and height were used to categorise BMI-for-age z-score and height and waist circumference (WC) to assess the abdominal obesity status using WHtR.

### Statistical analysis

Statistical processing and data analysis were performed using Microsoft Excel V.2016 and STATA V.16. Anthropometric data were converted to BMI-for-age z-score using the macro of the STATA igrowup package.^24^ Data were weighted to ensure representativeness. Frequencies, proportions and means were used to describe the results. Descriptive analysis was used to describe the socioeconomic, sociodemographic, anthropometric, health status and dietary characteristics of adolescents. Comparisons of mean values and prevalence were made by analysis of the Wald test. Bivariate analysis was initially performed to identify the factors associated with the dependent variables (underweight, overweight, obesity and abdominal obesity) in adolescents. Then, logistic regression models were used to examine the potential predictors of nutritional status. A value of p<0.05 was considered statistically significant for all statistical analyses. Model goodness-of-fit was performed using the Hosmer and Lemeshow test.

### Patient and public involvement

None.

## Results

**Figure 1:**
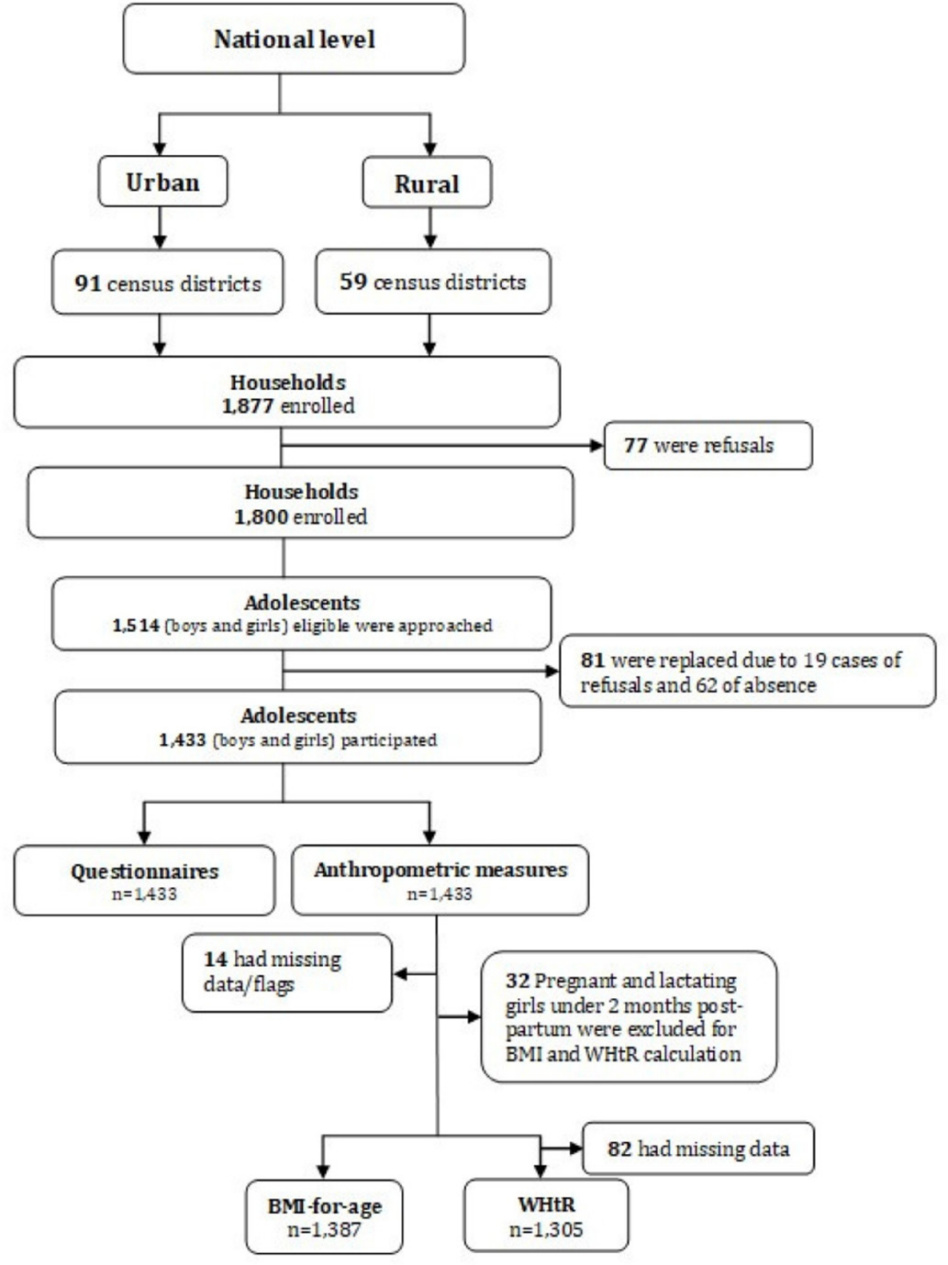
Study profile flow chart. BMI-for-age, body mass index-for-age; WHtR, waist-to-height ratio.

A total of 77 out of the 1800 households did not give their approval or were unavailable at the time of the survey. Out of 1514 eligible adolescents approached within these households, 1433 participated in the survey. The response rate of the study was 94.6% due to 19 cases of refusal and 62 cases of absence. BMI-for-age z-score and WHtR were calculated in 1387 and 1305 adolescents, respectively (figure 1).

### Demographic and socioeconomic-related characteristics

The mean age of the adolescents was 14.2±2.8 years. Moreover, 4 out of 10 adolescents were in the early adolescence stage, 38.7% in the middle stage and 17.9% in the late stage. Adolescents were mainly girls (53.4%). A higher proportion of adolescents were educated (79.6%) and 45.5% had reached over primary-level education. Overall, 18% of the adolescents lived in households belonging to the poorest wealth quintile and 24.6% of them to the richest (table 1). Most adolescents from the poorest and second wealth quintiles resided in rural areas (p<0.01).

**Table 1:** Demographic and socioeconomic-related characteristics of Senegalese adolescents

	National n=1433	Rural n=599	Urban n=834	P value
Age (years), M±SD	14.25±2.81	13.96±2.43	14.6±3.16	0.040
Age group (years), % (n)				0.002
10–14	52.7 (737)	57.1 (332)	47.0 (405)	
15–19	47.3 (696)	42.9 (267)	53.0 (429)	
Gender, % (n)				0.087
Boys	46.6 (625)	49.1 (287)	43.4 (338)	
Girls	53.4 (808)	50.9 (312)	56.6 (496)	
Stage of adolescence (years), % (n)				
Early 10–13	43.4 (604)	48.6 (284)	36.7 (320)	0.000
Intermediate 14–17	38.7 (559)	34.9 (208)	43.5 (351)	0.008
Late 18–19	17.9 (270)	16.5 (107)	19.8 (163)	0.178
Education, n (%)				
Yes	79.6 (1167)	71.1 (418)	90.5 (749)	0.000
Level of education, % (n)				
Primary	45.5 (519)	48.6 (192)	42.3 (327)	0.150
Medium	25.0 (321)	14.8 (66)	35.3 (255)	0.000
Secondary	10.2 (134)	5.3 (25)	15.2 (109)	0.000
Daara/non-formal education	10.4 (96)	18.3 (75)	2.5 (21)	0.000
Arabic school	7.3 (77)	11.5 (54)	3.0 (23)	0.002
Others	1.6 (20)	1.5 (6)	1.7 (14)	0.806
Socioeconomic status, % (n)				
Lowest	18.0 (281)	27.1 (196)	6.4 (85)	0.000
Second	17.3 (270)	20.0 (130)	13.9 (140)	0.009
Intermediate	19.6 (287)	21.3 (123)	17.6 (164)	0.132
Fourth	20.5 (290)	16.1 (86)	26.1 (204)	0.001
Highest	24.6 (305)	15.6 (64)	36.0 (241)	0.000

Values are means±SD; proportions (%) and number (n). P values represent differences between rural and urban adolescents using Wald test.

### Eating habits

Nearly 90% of Senegalese adolescents consumed three main meals daily and this trend was also observed in both rural and urban areas (table 2). Breakfast and lunch were more taken at home on weekdays in rural areas than urban areas (p<0.001). About 27% of adolescents ate outside the home, mainly purchasing food from street vendors and school canteens. Furthermore, 60.3% of adolescents reported snacking, with 55.4% of them at least once a day. This practice was significantly higher in urban areas (66.4%) than in rural areas (55.5%) (p<0.05). Over 74% of the adolescents reported having consumed dietary supplements in the month preceding the survey. Overall, 0.6% of the adolescents reported consuming alcoholic beverages, and less than 1% smoked.

**Table 2:** Eating habits of adolescents in Senegal

	National n=1433	Rural n=599	Urban n=834	P value
	% (n)	% (n)	% (n)	
Number of meals eaten/day	11.7 (164)	11.5 (61)	12.0 (103)	0.827
Less than three meals				
Meals taken at home on weekdays				
Breakfast	95.6 (1324)	98.1 (570)	92.4 (754)	0.000
Lunch	98.2 (1374)	99.9 (589)	96.0 (785)	0.000
Dinner	97.7 (1368)	97.8 (572)	97.7 (796)	0.900
Meals taken at home on weekends				
Breakfast	98.9 (1371)	99.2 (576)	98.5 (795)	0.238
Lunch	99.6 (1393)	99.7 (588)	99.4 (805)	0.356
Dinner	99.1 (1377)	99.7 (578)	98.5 (799)	0.028
Taking meals out of home				
Yes	26.7 (392)	20.4 (120)	34.8 (272)	0.000
Place where meals were taken out of home				
Stallholder/street vendor	12.7 (179)	11.2 (64)	14.6 (115)	0.241
School/service canteen	4.8 (75)	2.3 (10)	7.9 (65)	0.000
Small local eatery	2.7 (51)	1.6 (9)	4.1 (42)	0.008
Fast food	1.6 (34)	0.0 (0)	3.7 (34)	0.000
Modern restaurant	1.1 (25)	0.1 (1)	2.3 (24)	0.000
Others	8.1 (110)	7.8 (50)	8.5 (60)	0.680
Snacking				
Yes	60.3 (894)	55.5 (345)	66.4 (549)	0.011
Frequency of snacking				
At least once a day	54.2 (511)	49.6 (185)	59.0 (326)	0.087
4 to 6 times/week	25.6 (213)	26.8 (84)	24.2 (129)	0.562
1 to 3 times/week	17.3 (151)	20.8 (68)	13.7 (83)	0.080
1 to 3 times/month	2.5 (18)	2.8 (8)	2.2 (10)	0.715
Once a month	0.4 (1)	0.0 (0)	0.8 (1)	–
Alcohol drinking				
Yes	0.6 (16)	0.2 (3)	1.1 (13)	0.033
Smoking				
Yes	0.6 (7)	1.0 (6)	0.1 (1)	0.070
Consumption of dietary supplements				
During the survey	3.7 (72)	2.6 (19)	5.2 (53)	0.023
Last month before the survey	74.8 (55)	68.3 (13)	78.9 (42)	0.341

Values are proportions (%) and numbers (n); values of p represent differences between rural and urban areas using the Wald test.

### Dietary diversity

**Figure 2:**
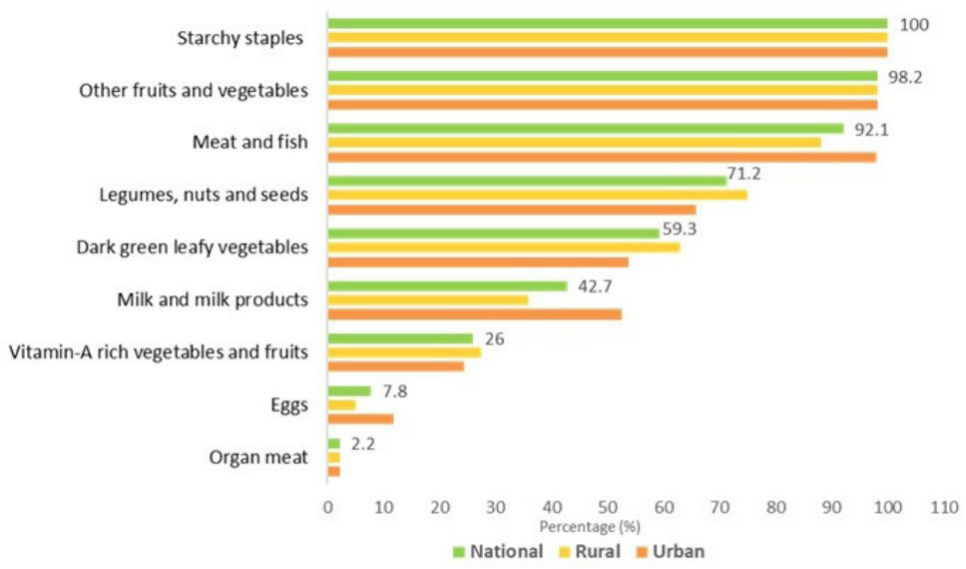
Consumption of nine food groups by adolescent boys aged 10–19 years and girls aged 10–14 years.

Starchy staple foods were widely consumed by adolescents. Most of them aged 10–19 years and girls aged 10–14 years reported having consumed other fruits and vegetables (98.2%), meat and fish (92.1%). The least consumed foods included organ meat (2.2%) and eggs (7.8%). However, rural adolescents consumed significantly more dark green leafy vegetables (p<0.05), legumes, nuts and seeds (p<0.05), whereas those living in urban areas ate more meat, poultry and fish (p<0.01), eggs (p<0.01) and milk, and milk products (p<0.01). For girls aged 15–19 years, the other major food group consumed in addition to starchy staples (99.5%) was the other vegetables group (97.2%) and the least consumed group was eggs (8.8%) (figures2  3).

**Figure 3:**
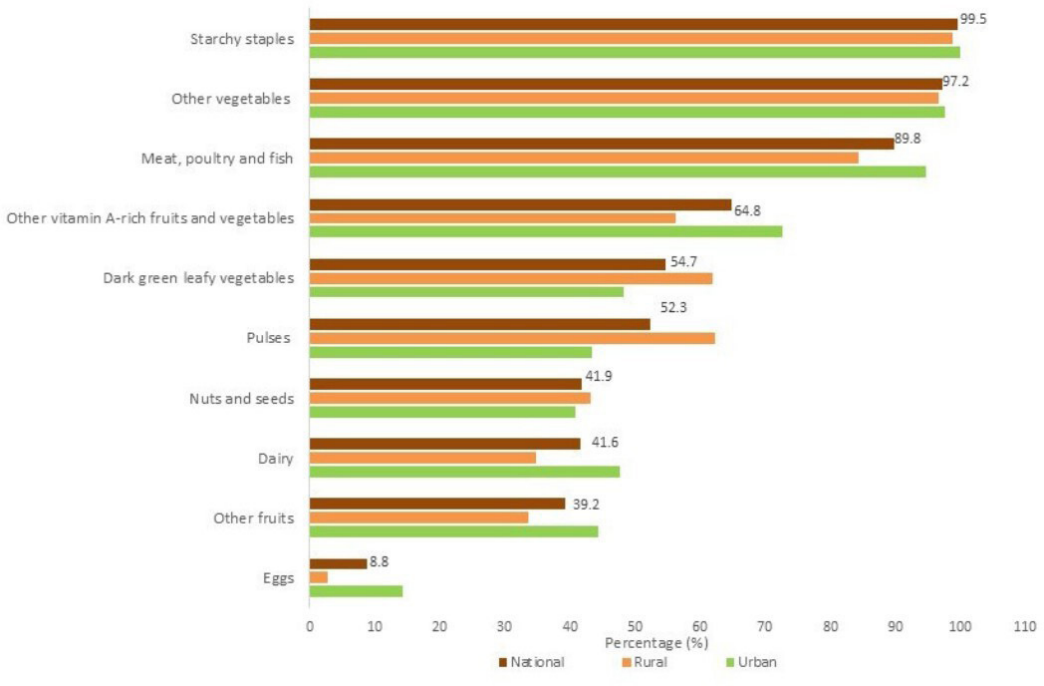
Consumption of 10 food groups by adolescent girls aged 15–19 years.

The mean DDS of adolescents was 5.23±1.28. The mean DDS in girls aged 15–19 years was 5.89 and 4.99 for the other adolescents (table 3). Around 72% of adolescents met the minimum dietary diversity (MDD). Nearly 16% of adolescent girls aged 15–19 years and 32.0% of other adolescents had not met the MDD.

**Table 3:** Dietary diversity of adolescents in Senegal

	All n=1433	Adolescent girls 15–19 years n=1028	Other adolescents* n=405
Dietary Diversity Score, M±SD	5.23±1.28	5.89±1.46	4.99±1.13
Adequate DD (≥5 food groups), % (n)	72.1 (1026)	83.9 (388)	68.0 (688)
Inadequate DD (<5 food groups), % (n)	27.9 (407)	16.1 (67)	32.0 (340)

Values are means±SD, proportions (%) and number (n).

* Other adolescents=adolescent boys aged 10–19 years and girls aged 10–14 years.

DD, dietary diversity of adolescents based on different food groups.

### Nutritional status

Nearly 23% of adolescents were underweight, 5.9% were overweight and obesity and abdominal obesity affected 1.8% and 5% of them, respectively (table 4). Underweight was significantly higher in rural (26.6%) than in urban areas (15.5%) (p<0.001). Boys were more affected than girls (p<0.001), and adolescents aged 10–14 years were more affected than older adolescents (p<0.05). Besides, overnutrition was significantly higher in urban areas compared with rural areas (p<0.05). Overweight and obesity affected 7.8% and 3.1% of adolescents in urban areas, respectively, and 6.8% of urban adolescents suffered from abdominal obesity. Girls were particularly suffering from overweight (9.4%) and abdominal obesity (8.5%) compared with boys (p<0.001). Overweight and obesity affected more adolescents living in the highest quintile (7.9% and 3.6%, respectively). Adolescents living within the highest wealth quintile were also more affected by abdominal obesity than others (p<0.05).

**Table 4:** Nutritional status of adolescents in Senegal

	BMI z-score, % (n)					
	N	Underweight	Overweight	Obesity	Abdominal obesity	
		n=287	n=79	n=30	N	n=64
National	1387	21.7 (287)	5.9 (79)	1.8 (30)	1305	5.0 (64)
Area of residence						
Rural	572	26.6 (155)	4.3 (19)	0.7 (5)	541	3.7 (16)
Urban	815	15.5 (132)	7.8 (60)	3.1 (25)	764	6.8 (48)
P value	–	< 0.001	< 0.05	0.004	–	0.047
Gender						
Boys	620	26.6 (160)	2.1 (17)	1.1 (8)	619	1.8 (14)
Girls	767	17.1 (127)	9.4 (62)	2.5 (22)	686	8.5 (50)
P value	–	< 0.001	< 0.001	0.091	–	0.000
Age (years)						
10–14	732	24.7 (180)	5.7 (39)	2.0 (16)	658	4.0 (24)
15–19	655	18.1 (107)	6.1 (40)	1.5 (14)	647	6.2 (40)
P value	–	< 0.05	0.804	0.473	–	0.229
Stage of adolescence (years)						
10–13	599	25.8 (157)	6.0 (35)	2.1 (12)	526	4.0 (20)
14–17	547	20.2 (97)	5.3 (28)	1.8 (14)	541	5.4 (28)
18–19	241	14.1 (33)	7.0 (16)	1.0 (4)	238	6.0 (16)
P value	–	< 0.01	0.797	0.414	–	0.465
Education						
Yes	1136	21.2 (229)	6.2 (69)	2.0 (27)	1068	5.6 (58)
No	251	23.6 (58)	4.4 (10)	1.1 (3)	237	2.7 (6)
P value	–	0.547	0.281	0.328	–	0.057
Wealth quintile						
Lowest	228	23.2 (65)	4.3 (11)	1.2 (4)	257	2.9 (10)
Second	292	26.0 (62)	4.8 (12)	0.5 (3)	250	3.1 (8)
Intermediate	279	19.8 (53)	3.7 (16)	0.6 (2)	263	1.8 (6)
Fourth	283	19.5 (52)	7.8 (19)	2.4 (6)	259	8.0 (16)
Highest	295	20.8 (55)	7.9 (21)	3.6 (15)	276	8.2 (24)
P value	–	0.473	0.270	0.078	–	0.010
Number of meals eaten per day						
Less than three meals	159	25.1 (31)	8.2 (12)	2.6 (7)	149	11.2 (17)
Three meals	1228	21.2 (256)	5.6 (67)	1.7 (23)	1256	4.2 (46)
P value	–	0.358	0.327	0.429	–	0.022
Snacking						
Yes	867	23.8 (189)	5.6 (46)	1.7 (16)	810	4.3 (35)
No	520	18.4 (98)	6.3 (33)	1.9 (14)	495	6.2 (29)
P value	–	0.043	0.724	0.770	–	0.257
Frequency of snacking						
At least once a day	495	22.5 (110	3.3 (19)	2.4 (11)	467	3.2 (18)
Four to six times/week	209	29.0 (53)	12.1 (19)	1.4 (4)	192	9.9 (6)
Once to three times/week	145	23.6 (25)	4.1 (8)	0.3 (1)	134	0.3 (1)
Once to three times/month	17	1.7 (1)	0.0 (1)	0.0 (1)	16	0 (0.0)
Once a month	1	0.0 (0)	0.0 (0)	0.0 (0)	1	0 (0.0)
P value	–	< 0.0001	< 0.0001	< 0.05	–	< 0.05

Values are proportions (%) and numbers (n); values of p represent differences using the Wald test.

BMI, body mass index.

### Factors associated with nutritional status

Multivariate logistic regression model factors that were independently associated with greater prevalence of underweight were boys, rural area and health status. The 10–14 years age group (OR=1.44; p<0.05), male gender (OR=1.74; p<0.001), skipping lunch daily (OR=2.28; p<0.01), snacking (OR=1.51; p<0.05), illness during the previous month (OR=1.78; p<0.01) and having diarrhoea (OR=4.86; p<0.05) increased the risk of being underweight. On the other hand, girl gender was a predictor of being overweight (OR=4.68; p<0.001) and suffering from abdominal obesity (OR=5.28; p<0.001). Urban adolescents were 59% more likely to be obese (p<0.05), whereas rural adolescents were more at risk of underweight (OR=1.91; p<0.01). Consuming dietary supplements had greater odds of being obese (OR=3.89; p<0.05). Adolescents who did not take breakfast daily had 4.5 times more risk of suffering from abdominal obesity (p<0.01) (table 5).

**Table 5:** Factors associated with nutritional status in adolescents in Senegal

	OR	95% CI	P value
	Underweight		
Area of residence			
Urban	1	Ref	
Rural	1.91	1.35 to 2.68	0.000
Sex			
Girls	1	Ref	
Boys	1.74	1.25 to 2.41	0.001
Age group (years)			
15–19	1	Ref	
10–14	1.44	1.00 to 2.07	0.049
Taking lunch daily			
Yes	1	Ref	
No	2.28	1.29 to 4.03	0.005
Snacking			
Yes	1.51	1.06 to 2.14	0.020
No	1	Ref	
Illness during the last month			
Yes	1.76	1.24 to 2.48	0.001
No	1	Ref	
Diarrhoea			
Yes	4.86	1.29 to 18.32	0.020
No	1	Ref	
	Overweight		
Gender			
Girls	4.68	2.39 to 9.16	0.000
Boys	1	Ref	
	Obesity		
Area			
Urban	4.08	1.36 to 12.25	0.013
Rural	1	Ref	
Dietary supplement consumption*			
Yes	3.89	1.11 to 13.65	0.034
No	1	Ref	
	Abdominal obesity		
Gender			
Girls	5.28	2.57 to 10.85	0.000
Boys	1	Ref	
Taking breakfast daily			
Yes	1	Ref	
No	4.55	2.10 to 9.85	0.000

P values represent p<0.05 for categorical variables using a regression logistic model.

* During the survey.

## Discussion

This study is the first nationwide study to examine sociodemographic, dietary and lifestyle associated factors for both underweight and overweight/obesity among Senegalese adolescents aged 10–19 years. The key findings of the study have demonstrated the existence of the double burden of malnutrition among adolescents in Senegal. The prevalence of underweight found in this study (21.7%) exceeds the latest trends revealed by the Global Nutrition Report^7^ and Fiorentino *et al*^18^ in Senegal. This prevalence was also higher than those found in surveys in Gambia,^28^ Ethiopia^29^ and India.^30^ These differences may primarily reflect the broader age range (10–19 years) and the anthropometric indices used. Indeed, most previous studies focused on adolescents aged 15–19 years. In addition, using WHO-recommended BMI-for-age rather than the BMI commonly used in previous studies may explain some differences, though no consensus has yet been established on anthropometric indicators.^9^ Our findings also revealed that adolescents living in rural areas were more likely to be affected by underweight than those in urban areas. This may be due to the poor living conditions in rural areas in Senegal and the limited access to health services.

Notably, similar findings were observed in Tanzania^31^ and India.^32^ In our study, the higher prevalence found in boys compared with girls was in line with those reported by several authors in African adolescents^33^ and among Indonesian adolescents.^34^ In addition, we found that adolescent boys were more exposed to underweight than girls. Mixed results and contrasting findings were reported by Anand and Sharma,^32^ in a systematic review in sub-Saharan Africa^35^ and in Denmark.^36^ The high prevalence of undernutrition among boys may be attributed to the difference in the physiological maturation period of adolescent boys and girls. Indeed, adolescent girls generally reach maturation earlier than boys, resulting in increased dietary requirements and energy expenditure. Another reason may be the distribution of body composition during adolescence, with boys having about twice the fat-free mass of girls.^37^,^39^

Our study reported a greater association between underweight and illness. Thus, being ill during the past 4 weeks and having diarrhoea were found to be predictive of adolescent underweight. In fact, diseases are immediate causes of malnutrition.

The study also revealed that skipping lunch regularly increased the risk of being underweight. This finding is consistent with the study conducted by Ali *et al*^40^ in Ethiopia and may be explained by the tendency of this habit to reduce dietary intake, thereby increasing the risk of underweight among adolescents. Snacking was found to be a common dietary habit. A higher proportion was also reported in Ghanaian high school adolescents (86.1%).^41^ Snacking may result from a preference for snacks or a dislike of available foods.^41^ Our study found that snacking predicted undernutrition, likely due to decreased food intake during main meals.^41^ In addition, adolescence is a period marked by specific dietary habits, especially snacking due to preferences and peer influence. Adolescents, especially in urban areas, often adopt snacking habits influenced by peers and long hours spent outside the home, which may result in skipped meals, particularly breakfast.

Besides, the prevalence of overweight was around 6% in our study. A similar and recent finding was reported in Ghana by Wiafe *et al.*^42^ Greater prevalences were found in previous studies carried out in some African countries.^43 44^ Trends at the global level showed that overweight has increased from 7.5% to 11.7% between 2000 and 2016^5^ and is low. The discrepancy between prevalences in these studies can be explained by the use of BMI in some studies and BMI-for-age in this study. BMI-for-age is known to be a better indicator than BMI in adolescents due to the fact that it takes account of these normal variations, providing a more accurate assessment of nutritional status.^45 46^ This study also highlighted overweight regarding gender and has shown that girls suffered more from overweight than boys, which agreed with previous reports in Dakar, Senegal.^19^ Some studies reported higher prevalence in boys rather than girls in north-eastern China.^47^ Interestingly, in our study, girls were found four times more likely to be overweight than boys, probably because fat mass increases more rapidly in girls^36^ and the limited opportunities for physical activity.^48^ On the other hand, our findings in obesity (1.8%) are also consistent with studies conducted in Senegal^19^ and Nigeria.^49^ A recent meta-analysis in seven countries of sub-Saharan Africa, that showed 3.2% of obesity, was also in contrast to our finding.^50^ This present study also highlighted gender disparities and higher prevalence of obesity in girls rather than boys, and the results were similar to previous findings reported in the Global Nutrition Report.^7^ We also found that adolescents living in urban areas were more likely to be obese than rural adolescents, probably due to the high consumption of fatty foods in urban environments characterised by a nutrition transition. Indeed, urbanisation is increasing the variety of retail food outlets available to consumers, especially adolescents, including bakeries, restaurants, supermarkets, street vendors and traditional open-air markets.^51^

Our study brings forward the information that consumption of dietary supplements was associated with higher odds of obesity. To our knowledge, there is no study that has clearly demonstrated the relationship between obesity and dietary supplement intake in adolescents. But this may be due to the fact that supplements increase the appetite and dietary intake of adolescents. Another finding in overnutrition in this study is related to abdominal obesity which affects 3.7% of adolescents. Abdominal obesity increases cardiometabolic risk,^52 53^ and the prevalence found in this study may be the result of food transition in Senegal, the high consumption of fatty foods and the lack of physical activity among adolescents. Greater rates were found in Nigeria (37.2%).^54^ Differences in prevalence can be explained by the difference in the indicators used. In fact, in some studies, waist-to-hip ratio (WHR) or WC were used whereas WHtR was used to assess abdominal obesity in our study. Even though there is still no consensus on an international WC threshold for abdominal obesity in children and adolescents, some studies clearly showed the superiority of using WHtR as an anthropometric indicator of regional fat distribution which is consistent with our finding.^26 55^ Furthermore, WHtR appears to be more closely associated with the risk of chronic diseases than WC and WHR, with an increased risk above 0.5.^56^

In this study, adolescent girls had 5.28 times more risk of suffering from abdominal obesity than boys. This is in line with results reported in South Africa.^57^ This difference in gender may be explained by the fact that abdominal obesity varies according to gender, is high in boys from age 8 years to 13 years, and high in girls from age 14 years to 17 years.^58 59^ In addition, another study carried out in Brazil showed that girls were more sedentary than boys, which may contribute to increased adiposity.^60^ In contrast, Rakić *et al* found that Serbian boys tend to show greater abdominal obesity than girls.^61^

Disparities observed in malnutrition among Senegalese adolescents based on the settings and gender can also be due to environmental, economic and sociocultural factors. In fact, rural adolescents lived in households with poor health and economic conditions and were more at risk of underweight compared with those living in urban areas. Besides, parental influence and body image perceptions can influence adolescents’ nutritional status. In their perception, adolescent boys often want to be thin or build up muscle mass, which is considered an expression of masculinity, while girls associate being overweight with beauty and wellness. Boys were shown to be more underweight than adolescent girls who prefer to eat more and practise less physical activity.

Healthy eating habits contribute to growth and better nutritional status, especially during adolescence. Snacking was found to be a more common dietary lifestyle in urban adolescents than in rural adolescents and can be attributed to consumption of meals outside the home, greater availability of foods from street vendors, restaurants and supermarkets, and non-healthy food marketing on television. This study also found that adolescents who did not take breakfast daily were more predictive of suffering from abdominal obesity compared with their peers. The reason might be that adolescents who used to skip this meal tend to engage in compensatory overeating at lunch and dinner.

This study revealed that no association was found between dietary diversity and malnutrition in adolescents. The dietary diversity of adolescents is critical for their nutritional status and reflects the diet quality. A poor diet is a risk factor for malnutrition and major non-communicable diseases.^62^ The present findings indicate that 72% of them had met the minimum dietary diversity. However, dietary diversity was not significantly associated with malnutrition. This can be attributed to the fact that DDS typically provides the number of food groups consumed, not to the quantity of nutrient content. Further, it can be due to some confounding factors in our regression model. Our findings also highlighted that Senegalese adolescents’ diet mainly comprised starchy foods such as rice and millet. Low consumption of animal-based foods such as eggs, meat and vitamin A-rich fruits and vegetables, found in this study, may be an issue regarding the importance of such food groups in the diets of adolescents. This could be explained by the fact that the consumption of eggs and meat is not frequent in Senegalese households. The low consumption of such foods could affect their growth or lead to malnutrition when adolescents’ dietary intake does not meet their nutritional requirements. In rural areas, the lower consumption of animal-based foods such as meat, poultry, fish, eggs and dairy products might be attributed to limited economic conditions, as well as the higher cost of those foods in Senegal. Globally, the double burden of malnutrition found in our study can be explained by the fact that there is no programme for the management of malnutrition among adolescents in Senegal. Many programmes and policies on malnutrition are mostly addressed to children and women of reproductive age.

### Strengths and limitations

This study has a number of strengths. It was conducted using a large sample of Senegalese adolescent boys and girls living in both rural and urban areas. Moreover, including adolescents aged 10–19 years, even those out of school, strengthens our study. The national representativeness of the study allows the generalisability of the findings in adolescents living in Senegal, also in the western African region.

Some limitations were found in this study. Its cross-sectional design establishes correlation but not causality, so the presence or otherwise of a causal relationship could not be established. Further studies are needed to clarify the causes of malnutrition among Senegalese adolescents from both rural and urban areas and according to gender. Despite this limitation, the associations presented in this study provide valuable insights on the predictive capacity of diet and health for adolescent nutritional risk. But it would be interesting to assess physical activity and the amount of food consumed and see the association with malnutrition.

## Conclusion

This study provides, for the first time, nutritional status and dietary patterns of Senegalese adolescents aged 10–19 years at the national level. The study found that underweight was prominent in adolescents, particularly in boys. Overweight and obesity require particular attention among girls and in urban areas. Therefore, greater priority must be given to adolescents in nutrition policies with targeted interventions implemented both at the community level health systems and within schools. Such interventions may include malnutrition management, school meal programmes and iron and folic acid supplementation. Additionally, promoting healthy environments and integrating nutrition education into school curricula and community programmes will contribute to improving adolescents’ nutritional status. Therefore, this requires an effective involvement of all stakeholders from different sectors through multisectoral strategic plans. Multisectoral collaboration, involving various sectors of health, education, agriculture and social protection, is essential for addressing the issue of malnutrition among adolescents. Such an approach recognises that the deep-rooted causes of malnutrition are multidimensional and require synergies to sustainably optimise the impact of nutrition policies on the health and well-being of adolescents.

## Data Availability

Data are available upon reasonable request.
