# Supplementary material for: Nutritional status and associated factors among adolescents in Senegal: a nationwide cross-sectional study
Source: BMJ Open. 2025 Oct 28;15(10):e095574. doi: 10.1136/bmjopen-2024-095574 (PMC12570916; doi:10.1136/bmjopen-2024-095574)
Supplement: Supplementary file 1 [file bmjopen-2024-095574.reviewer_comments.pdf]

PEER REVIEW HISTORY

BMJ Open publishes all reviews undertaken for accepted manuscripts. Reviewers are asked to complete a checklist review form and are provided with free text boxes to elaborate on their assessment. These free text comments are reproduced below.

ARTICLE DETAILS

Title (Provisional)

Nutritional status and associated factors among adolescents in Senegal: a nationwide cross-sectional study

Authors

SY, Mariame; Diouf, Adama; Badiane, Abdou; DIAGNE, Maty; Diagne, Abdoulaye; Idohou-Dossou, Nicole

---

VERSION 1 - REVIEW

**Reviewer**	**1**
**Name**	**Kurniawan, Adi Lukas**
**Affiliation**	**University of Bonn Center for Development Research**
**Date**	**14-Jan-2025**
**COI**	**None**

General Comments:

1. Significance and Contribution: The study addresses an important public health issue by investigating the double burden of malnutrition among adolescents in Senegal. The nationwide scope and inclusion of both undernutrition and overnutrition make this study a valuable contribution to the field.

2. Clarity and Structure: The manuscript is well-structured and follows the STROBE guidelines. The objectives, methods, results, and discussion are clearly presented, and the use of tables and figures enhances the readability of the findings.

3. Relevance: The findings are highly relevant for policymakers and public health practitioners in Senegal and other low- and middle-income countries facing similar challenges.

Specific comments and feedback:

1. Introduction

The introduction provides a strong rationale for the study. However, it would benefit from a more detailed explanation of why adolescents in Senegal have been relatively neglected in nutrition programs compared to other groups (e.g., children under five or pregnant women).

The global and regional context is well-described, but consider including more recent data or trends from West Africa to strengthen the justification for the study.

2. Methods

Study Design: The cross-sectional design is appropriate for the study objectives. However, the manuscript could elaborate on how the three-stage stratified sampling ensured representativeness across different regions of Senegal. Moreover, how were the 150 Census Districts selected, and how do they represent the diversity of Senegal's population?

Eligibility Criteria: The exclusion of adolescents with mental and physical disabilities is noted. Could the authors clarify how this exclusion might have impacted the generalizability of the findings?

Anthropometric Measurements: The use of WHO growth references and waist-to-height ratio (WHtR) is appropriate. However, the manuscript could provide more details on how measurement errors were minimized during data collection.

Dietary Diversity: The use of FAO guidelines for dietary diversity is commendable. However, the manuscript could clarify why different food group classifications were used for adolescent girls aged 15-19 years compared to other adolescents.

3. Results

Dietary Diversity: The finding that 72% of adolescents met the minimum dietary diversity is interesting. However, the manuscript could explore why dietary diversity did not correlate with malnutrition outcomes. Moreover, why were eggs and organ meats consumed so infrequently, and how might this impact the nutritional status of adolescents?

4. Discussion:

Interpretation of Findings: The discussion effectively interprets the results in the context of existing literature. However, the manuscript could delve deeper into the potential cultural, economic, and environmental factors driving the observed gender and urban-rural disparities.

Policy Implications: The recommendation to prioritize adolescents in national nutrition policies is well-founded. However, the manuscript could provide more specific suggestions for interventions (e.g., school-based nutrition programs, community outreach).

Limitations: The limitations are adequately discussed. However, the authors could elaborate on how the cross-sectional design limits causal inferences and suggest directions for future longitudinal studies.

5. Conclusion:

The conclusion is concise and aligns with the study objectives. However, it could emphasize the need for multisectoral collaboration (e.g., between health, education, and agriculture sectors) to address adolescent malnutrition.

Other questions for the authors:

1. Gender Disparities: What cultural or societal factors might explain the higher prevalence of underweight among boys and overweight/obesity among girls?

2. Urban-Rural Differences: Could the authors provide more insights into the specific dietary or lifestyle differences between urban and rural adolescents that might explain the observed disparities?

3. Policy Recommendations: What specific strategies do the authors suggest for integrating adolescent nutrition into existing national programs?

Reviewer 2

Name Nindya, Triska

Affiliation Faculty of Medicine Universitas Indonesia, Doctoral Program In Nutrition

Date 10-Jun-2025

COI None

This is well written article.

However, the authors need to check:

Introduction

1. Page 5 of 29, line 85-86 and 86-87 need references to justify the statement
2. Justify why you use WHtR for abdominal obesity compared to other indicators for adolescent (with supporting references)

Methods

1. Page 6 of 29, line 97-98: What do you mean by "wealth quintile dietary habits"?
2. Page 7 of 29, line 122: What do you mean by DSS? Please provide an abbreviation
3. Page 7 of 29, line 122: You assessed "foods consumed over the 24-hour recall period". Did all the adolescents had 24-hour recall during the weekdays? Or some had during the weekend? Since there is possibility that the consumed food might be differ during the weekdays and weekend
4. In your introduction, you mention about food insecurity and sedentary lifestyles as major contributor for undernutrition (line 68-69). However, in your methods you did not assess food insecurity and sedentary lifestyles. You need to more harmonize your introduction and methods
5. Page 7 of 29, line 138. What did you do to minimize the identified bias? Please, provide an explanation
6. Page 7 of 29, line 146-147: What do you mean by this "height also for categorize the abdominal obesity"? Height is not an indicator for abdominal obesity

Results

1. In all of the tables, if you did statistical analysis (p value) you need to mentioned statistical test in the footnote of the tables

Discussion

1. Page 18 of 29, Line 282-283, please provide justification from previous research related to urban environment and food transition

Conclusion

1. Line 321, typo of eating
2. From your research, it revealed that there was a relationship between obesity and dietary supplements. You mention in you recommendation that “need to control the dietary supplement intake. Is it possible from elaborate this for your research data? For example: type of supplement, duration, frequency and dose etc. Therefore, it will provide clearer recommendation about how to control dietary supplement intake

Limitation of the study

How about the bias that you have mention in your study? Can this also be a potential limitation of your study?

VERSION 1 - AUTHOR RESPONSE

**Reviewer: 1<br>Dr. Adi Lukas Kurniawan, University of Bonn Center for Development Research, Universitätsklinikum Heidelberg Heidelberg Institute of Global Health**	
Comments to the Author:<br>General Comments:	
1. Significance and Contribution: The study addresses an important public health issue by investigating the double burden of malnutrition among adolescents in Senegal. The nationwide scope and inclusion of both undernutrition and overnutrition make this study a valuable contribution to the field.<br><br>2. Clarity and Structure: The manuscript is well-structured and follows the STROBE guidelines. The objectives, methods, results, and discussion are clearly presented, and the use of tables and figures enhances the readability of the findings.<br><br>3. Relevance: The findings are highly relevant for policymakers and public health practitioners in Senegal and other low- and middle-income countries facing similar challenges.	Thank you for taking the time to review our paper.<br><br>We greatly appreciate your positive feedback and the strengths you point out.
Specific comments and feedback	
Introduction<br>The introduction provides a strong rationale for the study. However, it would benefit from a more detailed explanation of why adolescents in Senegal have been relatively neglected in nutrition programs compared to other groups (e.g., children under five or pregnant women).	Thank you for your suggestion.<br><br>Explanation of adolescent consideration in nutritional policies and programs was given, and revision was made.<br><br>“In Senegal, adolescent nutrition has been relatively neglected. Most of nutrition programs or interventions have prioritized children under 5 years and women of reproductive age, with little focus on adolescents. Despite the inclusion of girls aged 15 to 19 years within the women of

	reproductive age group, insufficient attention has been given to the nutritional knowledge and status of adolescents, even within reproductive health interventions. Indeed, the country has aligned its priorities and interventions with global challenges and priorities for years, focusing on women and children while nutrition considerations for adolescents has only emerged recently and remains weak. Therefore, there is no national program of malnutrition management for adolescents."<br><br>We added the following references:<br>• CNDN (Conseil National de Développement de la Nutrition). Plan stratégique multisectoriel de la nutrition. 2024-2028: rapport final. Avril 2024.<br>• Manga JS, Moubarac JC, Vandevijvere S, Diagne M, Kwadjode K, Thiam EHM, Nicole I Dossou, Ndiaye N, Diouf A. Évaluation des politiques publiques et des actions gouvernementales visant la création d'environnements alimentaires sains au Sénégal ; Rapport global. Université Cheikh Anta Diop, Université de Montréal, Ministère de la Santé et de l'Action Sociale (UCAD/UdeM/MSAS), 2019, 236 pages
The global and regional context is well-described but consider including more recent data or trends from West Africa to strengthen the justification for the study.	Thank you for the suggestion to include recent data.<br>However, we did not find specifically statistics or trends of malnutrition from West Africa. Data are available for Africa globally and it was already mentioned in the manuscript.
2. Methods<br>Study Design: The cross-sectional design is appropriate for the study objectives. However, the manuscript could elaborate on how the three-stage stratified sampling ensured representativeness across different regions of Senegal. Moreover, how were the 150 Census Districts selected, and how do they represent the diversity of Senegal's population?	Thank you for your comments and relevant suggestions.<br><br>As mentioned in the manuscript, data are representative at national level and in areas of residence (Urban/rural) at regional level. Representativeness at the regional level requires additional human and financial resources, as well as time to conduct such a study.<br><br>Procedure of the selection of the 150 Census Districts were explained in the "Study design and setting" section as follows:<br>"This nationwide study was conducted from July to November 2021. A probabilistic sampling method was employed using a three-stage stratified design. Census Districts (CDs) were selected as the primary sampling units. In the

	second stage, 12 households with at least one adolescent were randomly selected within each CD using a sampling interval of 10. In the third stage, one adolescent was randomly selected from the eligible adolescents living in selected households. Considering the proportion of children as the rarest target group, 1,800 households were selected. A total of 150 CDs were randomly selected from urban and rural areas of residence, 91 in urban areas and 59 in rural areas. A total of 1,433 adolescents aged 10-19 years were enrolled. This multistage sampling approach enabled the representation of adolescents across diverse geographic, cultural, and socio-economic contexts in Senegal.”
Eligibility Criteria: The exclusion of adolescents with mental and physical disabilities is noted. Could the authors clarify how this exclusion might have impacted the generalizability of the findings?	The study was conducted on adolescents, except those with mental and physical disabilities. Those adolescents may not be able to give free and informed consent, or their ability to participate independently in the survey may be reduced. The health conditions of individuals with mental and physical disabilities could also substantially affect their anthropometric measurements. Similarly, the tools used may not be adapted to their conditions. It is therefore more appropriate to conduct separate studies for these specific groups. Excluding these specific groups does not prevent the generalization or extrapolation of the results to adolescents, as they constitute an apparently healthy population.
Anthropometric Measurements: The use of WHO growth references and waist-to-height ratio (WHtR) is appropriate. However, the manuscript could provide more details on how measurement errors were minimized during data collection.	A revision was made in “Bias” section “Measurement errors were first minimized during the preparatory phase through comprehensive enumerator training, which included a standardization session to ensure the accuracy of anthropometric measurements. In addition, a pilot study was conducted to test and refine data collection tools and procedures. During data collection, errors were further minimized through close supervision. Data collected by well-trained enumerators (lead and assistant measurers) were checked in the field by team supervisors to ensure compliance with standard procedures. Moreover, daily feedback was provided, allowing for the

	correction of identified errors before leaving the census districts. Adolescents who had missing or outliers' data were also excluded from the statistical analysis."<br><br>It was revised in the manuscript as follows:<br><br>"According to FAO recommendations, since adolescent girls aged 15-19 years are considered in the 15-49 years age group of women of reproductive age, the 10 food groups were used to calculate the minimum dietary score for women (MDD-W). For the other adolescents consisted of boys aged 10-19 years and girls aged 10-14 years, the 9 groups were used to assess the DDS for individuals. The DDS for all adolescents were classified as adequate (minimum DDS) and inadequate using cutoff 5 food groups."
Dietary Diversity: The use of FAO guidelines for dietary diversity is commendable. However, the manuscript could clarify why different food group classifications were used for adolescent girls aged 15-19 years compared to other adolescents.	
3. Results<br>Dietary Diversity: The finding that 72% of adolescents met the minimum dietary diversity is interesting. However, the manuscript could explore why dietary diversity did not correlate with malnutrition outcomes. Moreover, why were eggs and organ meats consumed so infrequently, and how might this impact the nutritional status of adolescents?	Thank you for your valuable comment.<br><br>The manuscript now provides an added explanation.<br>"However, dietary diversity was not significantly associated with malnutrition. This can be attributed to the fact that dietary diversity score typically provides the number of food groups consumed not the quantity of nutrient content and to some confounding factors in our regression model."<br><br>Eggs and meat consumption was very scarce.<br>"Low consumption animal-based foods such as eggs, meat and vitamin A-rich fruits and vegetables found in this study may be an issue regarding the importance of such food groups in the diets of adolescents. This could be explained by the fact that the consumption eggs and meat is not frequent in Senegalese households. The low consumption of such foods could affect their growth or lead to malnutrition when adolescents' dietary intake does not meet their nutritional requirements. In rural areas, the lower consumption of animal-based foods such as meat, poultry, fish, eggs, and dairy products might be attributed to the limited economic conditions, as well as the higher cost of those foods in Senegal."
4. Discussion: Interpretation of Findings: The discussion effectively interprets the results in the context of existing literature. However, the manuscript could delve deeper into the potential	Thank you for pointing out this.<br><br>Cultural, economic, and environmental factors underlying gender and urban-rural disparities are

cultural, economic, and environmental factors driving the observed gender and urban-rural disparities.<br><br>Policy Implications: The recommendation to prioritize adolescents in national nutrition policies is well-founded. However, the manuscript could provide more specific suggestions for interventions (e.g., school-based nutrition programs, community outreach).<br><br>Limitations: The limitations are adequately discussed. However, the authors could elaborate on how the cross-sectional design limits causal inferences and suggest directions for future longitudinal studies.	comprehensively addressed in the manuscript on pages 18 and 19.<br><br>Thank you for your suggestion. We added it in the conclusion.<br>“Therefore, greater priority must be given to adolescents in nutrition policies with targeted interventions implemented both at the community level, health systems and within schools. Such interventions may include malnutrition management, school meal programs, iron and folic acid supplementation. Additionally, promoting healthy environments and integrating nutrition education into school curricula and community programs will contribute to improving adolescents’ nutritional status.”<br><br>Thank you for your comment. We added it to the manuscript.<br><br>“This cross-sectional design establishes correlation but not causality, so the presence or otherwise of a causal relationship could not be established. Further studies are needed to clarify the causes of malnutrition among Senegalese adolescents from both rural and urban areas and according to gender.”
5. Conclusion:<br>The conclusion is concise and aligns with the study objectives. However, it could emphasize the need for multisectoral collaboration (e.g., between health, education, and agriculture sectors) to address adolescent malnutrition.	Noted, we added this paragraph to emphasize this in the conclusion.<br><br>“Multisectoral collaboration, involving various sectors of health, education, and agriculture, social protection is essential for addressing the issue of malnutrition among adolescents. Such an approach recognizes that the deep-rooted causes of malnutrition are multidimensional and require synergies to sustainably optimize the impact of nutrition policies on the health and well-being of adolescents.”
Other questions for the authors:<br>1. Gender Disparities: What cultural or societal factors might explain the higher prevalence of underweight among boys and overweight/obesity among girls?	Thank you for your comment.<br><br>Gender Disparities were deeply discussed in the manuscript on page 18.<br>“Disparities observed in malnutrition among Senegalese adolescents based on their settings and gender can be also due to environmental, economic and socio-cultural factors. In fact, rural adolescents lived in households with poor health and economic conditions, and more at risk of underweight compared to those living in urban areas. Besides, the parental influence and body

	image perceptions can influence adolescents' nutritional status. In their perception, adolescent boys often want to be thin or build up muscle mass, which is considered as an expression of masculinity while girls associate being overweight with beauty and wellness. Boys were shown to be more underweight than adolescent girls who prefer to eat more and practice less physical activity."
2. Urban-Rural Differences: Could the authors provide more insights into the specific dietary or lifestyle differences between urban and rural adolescents that might explain the observed disparities?	Thank you for your comment.<br><br>Specific dietary or lifestyle discrepancies observed in rural and urban adolescents were better explained in 'Discussion 'section.<br>"Healthy eating habits contributes to growth and better nutritional status especially during adolescence. Snacking was found to be a more common dietary lifestyle in urban adolescents than rural adolescents and can be attributed to meals' consumption outside home, greater availability of foods from street vendors, restaurants and supermarkets, non-healthy food marketing on television."
3. Policy Recommendations: What specific strategies do the authors suggest for integrating adolescent nutrition into existing national programs?	Thank you for your comment.<br><br>Strategies for integrating adolescent nutrition into existing national programs were revised in the conclusion.<br>"Therefore, greater priority must be given to adolescents in nutrition policies with targeted interventions implemented both at the community level, health systems and within schools. Such interventions may include malnutrition management, school meal programs, iron and folic acid supplementation. Additionally, promoting healthy environments and integrating nutrition education into school curricula and community programs will contribute to improving adolescents' nutritional status."
**Reviewer: 2**<br>**Mrs. Triska Nindya, Faculty of Medicine Universitas Indonesia, Faculty of Public Health Universitas Airlangga**	
Comments to the Author:	
Introduction	

1. Page 5 of 29, line 85-86 and 86-87 need references to justify the statement	Thank you for your suggestion.<br><br>The following reference was added to justify the fact that ‘most of the studies in Senegal did not address the dietary habits and health status in adolescents, making it difficult to explore the whole adolescence target group findings.’<br><br>• Reference: Verstaeten R, Salm L, Diop L, Diatta A, Toure M. Adolescent Nutrition in West Africa: A Rapid Review of the Research Evidence. Dakar: IFPRI. April 2020.<br><br>Two references were added to justify that there is no national program for malnutrition management of adolescents in Senegal.<br><br>• CNDN (Conseil National de Développement de la Nutrition). Plan stratégique multisectoriel de la nutrition. 2024-2028: rapport final. Avril 2024.<br><br>• Manga JS, Moubarac JC, Vandevijvere S, Diagne M, Kwadjode K, Thiam EHM, Nicole I Dossou, Ndiaye N, Diouf A. Évaluation des politiques publiques et des actions gouvernementales visant la création d’environnements alimentaires sains au Sénégal; Rapport global. Université Cheikh Anta Diop, Université de Montréal, Ministère de la Santé et de l’Action Sociale (UCAD/UdeM/MSAS), 2019, 236 pages
2. Justify why you use WHtR for abdominal obesity compared to other indicators for adolescent (with supporting references)	Thank you for the suggestion.<br><br>The justification was revised.<br>“Even though there is still no consensus on an international WC threshold for abdominal obesity in children and adolescents (REF Lancet,), some studies clearly showed the superiority of using WHtR as an anthropometric indicator of regional fat distribution which is consistent with our finding. WHtR appears to be more closely associated with the risk of chronic diseases than WC, and WHR, with an increased risk above 0.5.”<br><br>The references were added as follows:<br>• Ashwell M, Gunn P, Gibson S. Waist-to-height ratio is a better screening tool than waist circumference and BMI for adult cardiometabolic risk factors: systematic review and meta-analysis. Obes Rev 2011;8:1-12.<br>• Hara M, Saito E, Iwata F, Okada T Harada K. Waist-to-height ratio is the best predictor of cardiovascular disease risk factors in Japanese

	schoolchildren. J Atheroscler Thromb 2002;9:127-132.
Methods	
1. Page 5 of 29, line 97-98: What do you mean by “wealth quintile dietary habits”?	It was revised.<br>wealth quintile dietary habits” was replaced by “wealth quintile”
2. Page 7 of 29, line 122: What do you mean by DSS? Please provide an abbreviation	Thank you for your comment.<br><br>DSS means ‘Dietary diversity score’ and the abbreviation has been already provided in the “Abstract section”.<br><br>It was added in the ‘Methods’ section as recommended.
3. Page 7 of 29, line 122: You assessed “foods consumed over the 24-hour recall period”.<br>Did all the adolescents had 24-hour recall during the weekdays? Or some had during the weekend? Since there is possibility that the consumed food might be differ during the weekdays and weekend	Thank you for your comment.<br><br>Due to the logistical complexity of conducting a nationwide survey, some adolescents completed the 24-hour dietary recall on weekdays, while others did so on weekends. There may be a potential variation in food consumption depending on whether the recall was conducted on a weekday or weekend.
4. In your introduction, you mention about food insecurity and sedentary lifestyles as major contributor for undernutrition (line 68-69). However, in your methods you did not assess food insecurity and sedentary lifestyles. You need to more harmonize your introduction and methods	Thank you for pointing this out.<br><br>The sentence “Food insecurity and sedentary lifestyles appear to be the major contributors to the substantial increase in undernutrition in low-income countries” as well as the reference were deleted.
5. Page 7 of 29, line 138. What did you do to minimize the identified bias? Please, provide an explanation	Thank you for your comment.<br><br>The ‘Bias’ section was revised.<br>“Measurement errors were first minimized during the preparatory phase through comprehensive enumerator training, which included a standardization session to ensure the accuracy of anthropometric measurements. In addition, a pilot study was conducted to test and refine data collection tools and procedures. During data collection, errors were further minimized through close supervision. Data collected by well-trained enumerators (lead and assistant measurers) were checked in the field by team supervisors to ensure compliance with standard procedures. Moreover, daily feedback was provided, allowing for the correction of identified errors before leaving the census districts.”

6. Page 7 of 29, line 146-147: What do you mean by this “height also for categorize the abdominal obesity”? Height is not an indicator for abdominal obesity	Thank you for the comment.<br><br>It was a mistake. The correction was done as follows:<br><br>“BMI-for-age z-score and height and waist circumference to assess the abdominal obesity status using WHtR.”
Results	
1. In all of the tables, if you did statistical analysis (p value) you need to mention statistical test in the footnote of the tables	Thank you for your comment.<br><br>Revision was done. In all tables, statistical tests were mentioned in the footnote.
Discussion	
1. Page 18 of 29, Line 282-283, please provide justification from previous research related to urban environment and food transition	The justification was provided as well as the reference the reference as follows:<br>“We also found that adolescents living in urban areas were more likely to be obese than rural adolescents, probably due to the high consumption of fatty foods in urban environment characterized by a nutrition transition. Indeed, urbanization is increasing the variety of retail food outlets available to consumers especially adolescents, including bakeries, restaurants, independent and chain supermarkets, street vendors, and traditional open-air markets.<br><br>• Thériault, V. et al. Diet transformations and changing food environments in the Sahel and West Africa. Sahel and West Africa Club (OECD/SWAC), West African Papers 2024; No. 45, OECD Publishing Paris.
Conclusion	
1. Line 321, typo of eatings	Thank you for your comment.<br><br>The correction was performedand replace by “dietary patterns” on page 20 in line 392.
2. From your research, it revealed that there was a relationship between obesity and dietary supplements. You mention in your recommendation that “need to control the dietary supplement intake. Is it possible to elaborate this for your research data? For example: type of supplement, duration, frequency and dose etc. Therefore, it will provide a clearer recommendation about how to control dietary supplement intake	Thank you for this important suggestion and this careful reading of the paper.<br><br>It would be interesting to know more about dietary supplement intake to better refine the recommendation on controlling dietary supplement intake. However, we have not gone in-depth on the issue. We only collected complementary data (type) from adolescents who consumed dietary supplements during the month before the survey, not during the survey.
Limitation of the study	

How about the bias that you have mentioned in your study? Can this also be a potential limitation of your study?	Thank you for the comment.<br><br>Bias that were mentioned cannot be a potential limitation and as revised in "Bias" section, all necessary measures were taken to ensure that biases were minimized.
Additional questions	
Please ensure the accuracy of information relating to your article, including author names and affiliations, title, abstract and required statements (e.g. competing interests, contributorship, funding).<br>Please check that this information has been entered correctly and has been updated as appropriate.	Thank you for your suggestion.<br>Updates of information relating to all the sections of the article were made.
Please ensure that all ORCID IDs have been added to co-authors' ScholarOne accounts before completing your submission. Note that only the individual user can add ORCID IDs to their account and the corresponding author is unable to add this to co-authors' accounts on their behalf. Additionally, BMJ is unable to add ORCID IDs to users accounts on their behalf. ORCID IDs should be linked to their ScholarOne account prior to acceptance. BMJ cannot add ORCID IDs during the production process or after publication.	Thank you for the useful suggestion.<br><br>However, ORCID IDs for co-authors have not been added. There is an issue for adding it. But we would like to add it.
COI statements:	
Reviewer: 1<br>If you have selected 'Yes' above, please provide details of any competing interests.: Not applicable.<br><br>Reviewer: 2<br>If you have selected 'Yes' above, please provide details of any competing interests.: Not applicable.	Thank you for your comment. It was revised.<br><br>"Competing interests<br>Not applicable"
Revision added by authors	
Table 1: SD values were corrected<br>The values in the table were the standard error values. It was revised by adding standard deviation values.	
Table 3: the word "food" in the title of the table was corrected<br>"Table 3 "Dietary diversity of adolescents" instead of "Table 3 Dietary diversity food of adolescents"	
In figure 1, we included the urban and rural settings.	

VERSION 2 - REVIEW

**Reviewer**	**2**
**Name**	**Nindya, Triska**
**Affiliation**	**Faculty of Medicine Universitas Indonesia, Doctoral Program In Nutrition**
**Date**	**25-Aug-2025**
**COI**	

The author(s) already responded to all of the comments, provided great explanation and revised the manuscript. Thank you very much

VERSION 2 - AUTHOR RESPONSE

Reviewer: 2<br>Mrs. Triska Nindya, Faculty of Medicine Universitas Indonesia, Faculty of Public Health Universitas Airlangga<br>Comments to the Author:	
The author(s) already responded to all of the comments, provided great explanation and revised the manuscript. Thank you very much.	Thank you very much for your comment. We greatly appreciate it.
If you have selected 'Yes' above, please provide details of any competing interests.: Not applicable	It was revised.
